# Comparison of the effects of high dietary iron levels on bone microarchitecture responses in the mouse strains 129/Sv and C57BL/6J

**DOI:** 10.1038/s41598-024-55303-2

**Published:** 2024-02-28

**Authors:** Maria G. Ledesma-Colunga, Vanessa Passin, Maja Vujic Spasic, Lorenz C. Hofbauer, Ulrike Baschant, Martina Rauner

**Affiliations:** 1 Department of Medicine III & Center for Healthy Aging, Medical Faculty and University Hospital Carl Gustav Carus, Dresden University of Technology, Dresden, Germany; 2 Institute of Comparative Molecular Endocrinology, Ulm University, Ulm, Germany

**Keywords:** Iron-rich diet, Bone loss, Mouse strains, 129/Sv, C57BL/6J, Cell biology, Endocrinology

## Abstract

Iron is an essential nutrient for all living organisms. Both iron deficiency and excess can be harmful. Bone, a highly metabolic active organ, is particularly sensitive to fluctuations in iron levels. In this study, we investigated the effects of dietary iron overload on bone homeostasis with a specific focus on two frequently utilized mouse strains: 129/Sv and C57BL/6J. Our findings revealed that after 6 weeks on an iron-rich diet, 129/Sv mice exhibited a decrease in trabecular and cortical bone density in both vertebral and femoral bones, which was linked to reduced bone turnover. In contrast, there was no evidence of bone changes associated with iron overload in age-matched C57BL/6J mice. Interestingly, 129/Sv mice exposed to an iron-rich diet during their prenatal development were protected from iron-induced bone loss, suggesting the presence of potential adaptive mechanisms. Overall, our study underscores the critical role of genetic background in modulating the effects of iron overload on bone health. This should be considered when studying effects of iron on bone.

## Introduction

Iron is an essential dietary nutrient for humans and other mammals^1^. Insufficient iron levels can lead to restricted erythropoiesis and anemia, while excessive iron accumulation can result in the generation of reactive oxygen species (ROS), causing cellular and organ damage^2^. To maintain iron balance, the liver serves as the primary site for iron storage and plays a pivotal role in the regulation of iron levels in the body through the production of hepcidin. Hepcidin, in turn, modulates plasma iron concentrations and tissue iron distribution by interacting with its receptor, ferroportin, an iron-exporting protein^1,3^.

Amongst various organs affected by iron overload, bone is particularly susceptible to alterations in iron levels^4^ as bone weakening, increased risk of fractures, and osteoporosis are often manifested in disorders associated with iron accumulation^5–8^. However, it remains uncertain whether these manifestations are primarily due to the direct impact of iron on bone or if they result from concurrent underlying comorbidities that could potentially influence bone metabolism.

Iron overload and its bone-related complications have primarily been investigated in preclinical animal models of hereditary hemochromatosis. This is mainly because these models exhibit concurrent tissue iron overload. However, it is important to note that these animal models exhibit a wide range of bone phenotypes, which can likely be attributed to the patterns of iron overload, the resulting effects on hepcidin regulation, but also to the specific intrinsic roles of the proteins affected by the genetic mutations. For instance, mice lacking the *Hamp* gene^9^ or harboring the *Fpn*^*C326S*^ mutation^10^ have shown reduced bone mass. However, the impact of *Hfe* deficiency on bone status remains a topic of debate^11,12^, and the effect of *Hjv* mutation has yet to be determined. Studies on mice lacking *Tfr2* have revealed bone-cell intrinsic functions, leading to a phenotype characterized by high bone mass, despite iron overload^13^.

Additionally, mouse models of iron overload achieved through exogenous administration of iron (in form of iron dextran, ferric ammonium citrate, and ferric nitriloacetate) that result in increased systemic iron stores have reported an osteoporotic phenotype characterized by increased bone resorption and oxidative stress^14–17^. However, not all the studies have shown effects on bone metabolism, and the variability of iron loading is highly dependent on the hemochromatosis mutation/type, duration and type of iron used, including studied mouse strain^3,18,19^.

In this study, we explored the effect of dietary iron overload (mice fed with a diet containing 2% of carbonyl iron) on the bone status in 129/Sv and C57BL/6J, two mouse strains frequently used in iron and bone research^20–22^. Our findings revealed that 129/Sv mice exhibited bone alterations, characterized by trabecular bone loss, thinner cortices, and decreased bone formation. In contrast, despite inducing systemic iron overload in our experimental setting, C57BL/6J mice are rather resilient and do not develop bone abnormalities. This study sheds light on the differential effects of dietary iron overload on bone metabolism in two inbred mouse strains, emphasizing the importance of strain selection in bone- and iron-related research.

## Materials and methods

### Mice, diets, and experimental design

The animal procedures were conducted in compliance with the guidelines of the institutional animal care committee and in adherence to the ARRIVE guidelines. Furthermore, all experimental protocols were approved by the Landesdirektion Sachsen (TVV 20/2020). All mice were purchased from Janvier Laboratory (France) a week before initiating the experiments.

Male 129/Sv mice were housed under a 12 h light/dark cycle and a controlled temperature (23 °C), with food (standard chow diet containing 176 mg iron/kg, Ssniff V1534-3) and water ad libitum. At the age of 6 weeks, mice (n = 7–8 per group) were assigned to either a control chow diet (CD; Ssniff E15510-24 supplemented with 200 mg of iron/kg) or an iron rich diet (IRD; Ssniff E15510-24 supplemented with 20,000 mg carbonyl iron/kg) for 6 weeks.

Similarly, C57BL6/J mice were maintained in a 12 h light/dark cycle at a controlled temperature (23 °C), and provided unrestricted access to water and food. At 6 weeks of age, young C57BL/6J mice (n = 7–8 per group) were divided in two groups and fed either a CD or an IRD for a duration of 6 weeks. Additionally, adult C57BL/6J mice (12 weeks old, n = 7–8 per group) were placed on a CD or an IRD for 6 or 12 weeks.

In a separate setting, 129/Sv mice in parental cages were kept in a 12 h light/dark cycle at a controlled temperature (23 °C), water ad libitum*,* and with free access to a CD or an IRD as previously described. These diets were given three weeks prior to pregnancy and maintained throughout pregnancy, with the offspring receiving the same diet as the dams. At 4 weeks of age, mice from each diet were further divided into two subgroups: one fed a CD (n = 8–12) the other fed an IRD (n = 8–12) until they reached 12 weeks of age. Following the conclusion of dietary maintenance, all mice were subjected to anesthesia using a combination of ketamine (100 mg/kg) and xylazine (10 mg/kg) based on their body weight. Subsequently, blood was obtained through a cardiac puncture. To euthanize the animals, cervical dislocation was performed while they were still under anesthesia, and their tissues were collected for subsequent analysis.

### Micro-computer tomography

µCT (vivaCT40, Scanco Medical) was performed on the excised femur and L4-vertebral body with an isotropic voxel size of 10.5 µm (70-kVp, 114 µA, and 200 ms integration time) and the threshold for bone set to 220 mg/HA/cm^3^. All scanned regions of the femora and vertebrae consisted of 600 slices. For the femur, manual contouring was employed to isolate the trabecular bone compartment (metaphysis, extending away from the growth plate), while in the vertebra, evaluation was focused around the center. For the cortical bone compartment was analyzed in the mid-shaft (diaphysis region midway between femoral head and distal condyles). Each compartment included regions spanning 100 slices. Established protocols from Scanco Medical were applied for the analysis. All µCT parameters were reported according to international guidelines^23^. Finally, the images were loaded for 3D visualization in the data viewer program supplied with the instrument.

### Hematological parameters and systemic iron measurements

To evaluate hematological parameters, a blood sample was obtained using an EDTA tube and subsequently diluted at a 1:3 ratio with NaCl 0.9%. This diluted sample was then subjected to analysis using the automated cell counter XN-1000 (Sysmex). For the assessment of serum iron levels, blood was collected in a non-coated plastic tube and subsequently centrifuged at 3000 rpm for 20 min. The resulting serum was utilized to determine serum iron concentration (SFBC) and unsaturated iron binding capacity (UIBC), employing colorimetric assays with the SFBC and UIBC kits (BioLabo). Transferrin saturation was calculated using the formula: (SFBC/(SFBC + UIBC)) × 100. Non-heme iron content in the liver and tibial shaft (when specified with or without bone marrow) was assessed using the bathophenanthroline colorimetric method in dried tissue^24^. Values are reported as µg/g of dry weight tissue.

### Serum bone markers

Serum levels of pro-collagen type 1 N-terminal peptide (PINP) and C-terminal telopeptide of type I collagen (CTX-I), which serve as markers of bone turnover, were determined using ELISAs according to the manufacturer`s protocol (Immunodiagnostics).

### RNA isolation, reverse-transcription and quantitative real-time PCR

Total RNA was extracted using Trizol (Invitrogen) from liver or frozen femoral shaft (when specified with or without bone marrow) that were ground with a mortar and pestle containing liquid nitrogen. Subsequently, 1 µg of total RNA underwent reverse transcription using M-MVL RT (H-) Point Mutant (Promega). Detection and quantification of PCR products were carried out through SYBR green-based real-time PCR, following a standard protocol from Promega. Oligonucleotide sequences (Sigma-Aldrich) utilized in the study are detailed in Suppl. Table 1. The PCR amplifications were analyzed using the 2^−ΔΔCT^ method, with cycle thresholds (CT) normalized to the housekeeping gene *Actb* to determine the mRNA levels of interest.

### Bone histology and histomorphometry

The fourth vertebral bodies were immersed in a 4% PBS-buffered paraformaldehyde solution for 48 h. Following this fixation, samples were dehydrated through a progressive ethanol series and subsequently embedded in paraffin. Subsequently, 2-μm-thick tissue sections were prepared and stained using tartrate-resistant acid phosphatase (TRAP). Evaluation of osteoblasts, osteoclasts, and osteocytes per bone perimeter, as well as osteoblasts and osteoclasts per bone surface in the stained sections, was performed using the Osteomeasure Analysis System (Osteometrics).

### Statistical analysis

Statistical analysis encompassed tests for assessing normality and equal variance. The Grubb`s test was employed to detect and remove outliers, followed by the recalculation of group means and standard deviations. When the conditions of normality and equal variance were met, comparisons between groups were made using a two-tailed Student's *t*-test (parametric). All statistical analyses were carried out using GraphPad Prism 9.0 (GraphPad Software Inc.). The data are presented as the mean ± standard deviation (SD), and significance levels were denoted as values of *P < 0.05, **P < 0.01, or ***P < 0.001.

## Results

### Dietary iron overload causes bone loss in male 129/Sv mice

**Figure 1:**
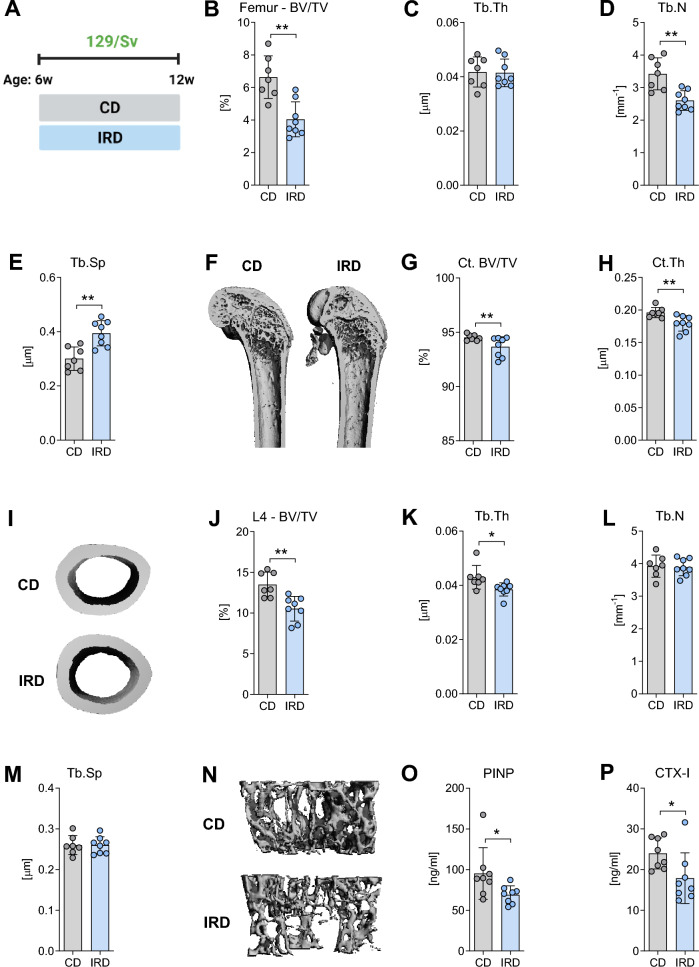
Trabecular and cortical bone morphology of the femora and vertebrae of 129/Sv mice fed a control diet or an iron-rich diet for 6 weeks. (**A**) Illustration depicting the experimental setup for inducing dietary iron overload. At 6 weeks of age mice were divided into two groups and fed either a control diet (CD) or an iron-rich diet (IRD) until 12 weeks of age. Trabecular bone parameters in the (**B**–**F**) femur and (**J**–**N**) L4-vertebra were assessed including (**B**, **J**) bone volume per total volume (BV/TV), (**C**, **K**) trabecular thickness (Tb.Th), (**D**,** L**) trabecular number (Tb.N), and (**E**, **M**) trabecular separation (Tb.Sp). (**F**, **N**) Representative 3D rendering of trabecular bone from the femur and L4-vertebra. (**G**) Cortical bone volume (Ct. BV/TV) and (**H**) cortical thickness (Ct.Th) were analyzed at the midshaft of the femur. (**I**) Representative 3D rendering of mid-diaphysis cortical bone from the femora. Serum levels of (**O**) pro-collagen type 1 N-terminal peptide (PINP) and (**P**) C-terminal telopeptide of type I collagen (CTX-I) were measured. Data are presented as mean ± SD (n = 7–8 per group). Each symbol represents an individual animal. Statistics were calculated using Student´s* t*-test. *P < 0.05, **P < 0.01, ***P < 0.001.

To assess the susceptibility of 129/Sv mice, which are broadly used in iron research^20–22^, to iron-induced bone loss, we employed an established model of dietary iron overload^25^. Mice were divided into two groups at 6 weeks of age: one group was fed a CD containing 200 mg of iron/kg, while the other group was fed an IRD containing 20,000 mg of iron/kg. The dietary regimen was maintained for a period of 6 weeks as depicted in Fig. 1A.

Our results indicate that 129/Sv mice, when given an IRD, exhibited a significant increase in liver iron content as well as the mRNA expression of *Hamp* compared to those on the CD (Suppl. Table 2). However, no significant alterations were observed in the content of iron in bone/bone marrow tissues. (Suppl. Table 2). In addition, we investigated the impact of iron overload on the expression of iron-related genes in bone tissue. Although the mRNA expression of *Hfe*, *Slc40a1*, and *Hamp* remained unchanged, a significant upregulation in the levels of *Tfr1* in bone from mice fed the IRD was observed (Suppl. Fig. 1).

To assess the impact of IRD on bone microarchitecture, we conducted µCT scans on both femur and fourth vertebral body. The analysis of trabecular bone at the distal femur showed that 129/Sv mice consuming the IRD displayed a notable reduction in bone volume when compared to their CD-fed counterparts (Fig. 1B). Moreover, although the trabecular thickness remained relative consistent (Fig. 1C), there was a significant decrease in trabecular numbers (Fig. 1D), leading to an increase in trabecular separation (Fig. 1E,F). In addition, mice fed the IRD also exhibited reduced cortical bone volume and thinner cortices compared to those fed a CD (Fig. 1G,H,I). Representative 3D rendering images of trabecular and cortical bone are depicted in Fig. 1F and I, respectively.

At the vertebral body, there was a notable decrease in the bone volume in mice fed an IRD compared to those on a CD (Fig. 1J). Consistently, trabecular thickness was reduced (Fig. 1K). However, no significant changes were observed in the number of trabeculae (Fig. 1L), or trabecular separation (Fig. 1M). Representative 3D rendering images are illustrated in Fig. 1N.

To assess the potential impact of an imbalance between bone resorption and bone formation processes on bone mass, we conducted evaluations encompassing plasma levels of bone turnover markers, dynamic histomorphometry and mRNA expression of osteoblast and osteoclast marker genes. Notably, levels of the bone formation marker PINP were significantly reduced in the serum from 129/Sv mice fed an IRD in comparison to the CD group (Fig. 1O). In addition, the circulating levels of the bone resorption marker CTX-I were also significantly reduced in mice fed an IRD (Fig. 1P). Although the parameters of bone remodeling assessed in TRAP-stained L4-vertebra were comparable between mice fed a CD or an IRD, we observed a significant upregulation of the mRNA expression of the osteoblast markers *Sp7*, *Runx2*, and *Postn*, while *Spp1* was decreased. Similarly, the expression of the osteoclast markers *Nfatc1*, *Oscar*, and *Acp5* in bone/bone marrow tissue was elevated, while *Ctsk* expression was significantly reduced (Suppl. Fig. 2). Taken together, these data suggest that dietary iron supplementation has a deleterious effect on bone metabolism by decreasing bone turnover.

### Dietary iron overload does not impair bone microarchitecture in young or adult male C57BL/6J mice

To explore whether the genetic background plays a role in the variation of iron levels among different mouse strains and how this affects bone characteristics, we conducted an analysis focusing on the consequences of iron overload on bone microarchitecture in C57BL/6J mice.

**Figure 2:**
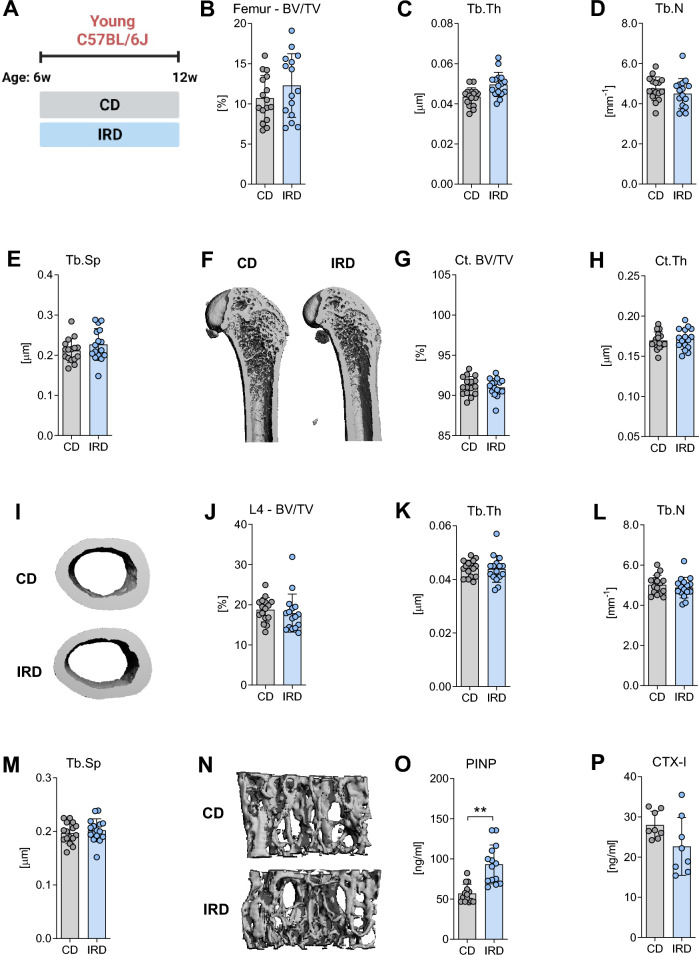
Trabecular and cortical bone morphology of the femora and vertebrae of young C57BL/6J mice fed a control diet or an iron-rich diet for 6 weeks. (**A**) Schematic representation of the experimental setup for inducing dietary iron overload. At 6 weeks of age mice were divided into two groups and fed either a control diet (CD) or an iron-rich diet (IRD) until 12 weeks of age. Trabecular bone parameters in the (**B**–**F**) femur and (**J**–**N**) L4-vertebra were assessed including (**B**, **J**) bone volume per total volume (BV/TV), (**C**, **K**) trabecular thickness (Tb. Th), (**D**, **L**) trabecular number (Tb.N), and (**E**, **M**) trabecular separation (Tb.Sp) were assessed in the femur and L4-vertebra. (**F**, **N**) Representative 3D rendering of trabecular bone from the femur and L4-verterbra. (**G**) Cortical bone volume (Ct. BV/TV) and (**H**) cortical thickness (Ct.Th) were analyzed at the mid-shaft of the femur. (**I**) Representative 3D rendering of mid-diaphysis cortical bone from the femora. Serum levels of (**O**) pro-collagen type 1 N-terminal peptide (PINP) and (**P**) C-terminal telopeptide of type I collagen (CTX-I) were also measured. Data are presented as mean ± SD (n = 8–14 per group). Each symbol represents an individual animal. Statistics were calculated using Student’s* t*-test. *P < 0.05, **P < 0.01, ***P < 0.001.

To ensure compatibility with our prior study involving 129/Sv mice, we subjected C57BL/6J to a similar experimental protocol. In brief, at 6 weeks of age, C57BL/6J mice were divided into two groups: one group received a CD containing 200 mg of iron/kg, while the other group was given an IRD containing 20,000 mg of iron/kg. This dietary regimen was maintained for a period of 6 weeks, as shown in Fig. 2A. After this specified period, we observe a substantial increase in hepatic iron content and increased mRNA levels of *Hamp* on the IRD compared to CD (Suppl. Table 3). Nevertheless, no significant changes were detected in in the iron content within bone/bone marrow tissues (Suppl. Table 3) or in the expression of the iron-related genes such as *Hfe*, *Tfr1*, *Slc40a1*, and *Hamp* in bone/bone marrow tissues (Suppl. Fig. 3). Similarly, no significant alterations were noted when comparing hematological or systemic iron parameters among diets (Suppl. Table 3). Moreover, we did not observe any significant effects on trabecular bone microarchitecture (Fig. 2B–F) or cortical morphology (Fig. 2G–I) within the femur after 6 weeks on IRD. These results remained consistent at the vertebral body (Fig. 2J–N). In addition, to investigate the effects of iron on osteoblast activity in vivo we assessed the levels of PINP in serum. As shown in Fig. 2O, a significant increase of PINP was observed in mice fed with the IRD. Additionally, to evaluate osteoclast activity, we measured CTX-I levels, but no significant effect was observed after 6 weeks of dietary intervention (Fig. 2P). No changes were observed in bone remodeling parameters or the expression of osteoblast marker genes (Suppl. Fig. 4). Notably, only the expression of the osteoclast marker genes *Oscar*, *Acp5*, and *Ctsk* was significantly upregulated in IRD-fed mice compared to CD mice.

**Figure 3:**
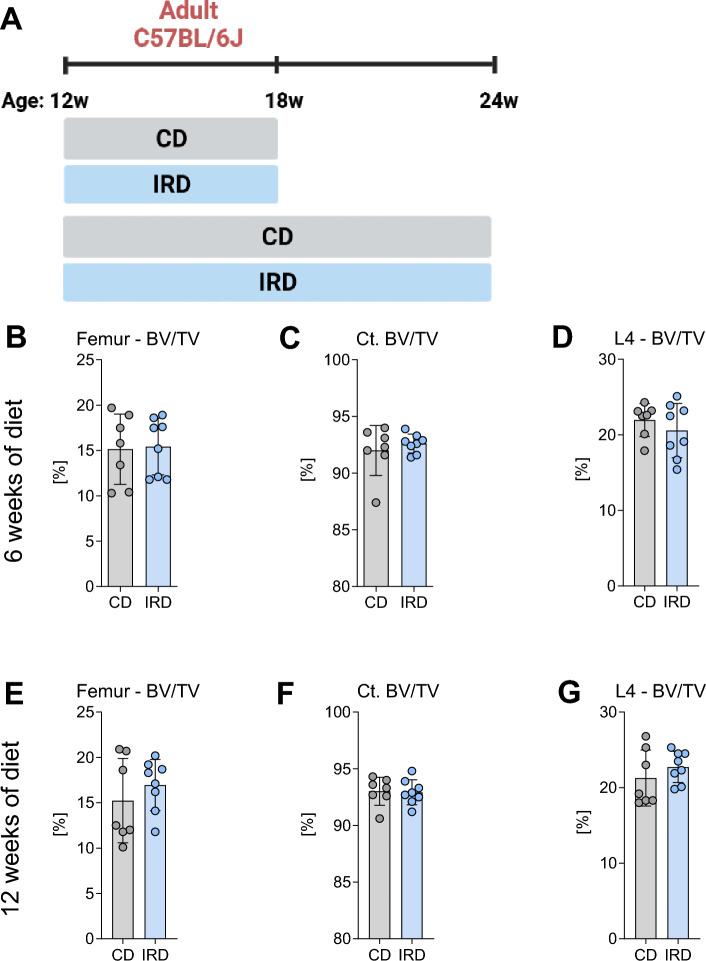
Trabecular and cortical bone density of the femora and vertebrae of adult C57BL/6J mice fed a control diet or an iron-rich diet for 6 or 12 weeks. (**A**) Schematic representation of the experimental setup for inducing dietary iron overload. At 12 weeks of age mice were divided into two groups and fed a control diet (CD) or an iron-rich diet (IRD) during 6 or 12 weeks. Trabecular bone volume per total volume (BV/TV) in (**B**, **D**) femur and (**D**, **G**) L4-vertebral body after 6 or 12 weeks of diet (18 weeks and 24 weeks of age, respectively). (**C**, **F**) Cortical bone volume (Ct. BV/TV) was analyzed at the midshaft of the femur. Data are presented as mean ± SD (n = 7–8 per group). Each symbol represents an individual animal. Statistics were calculated using Student’s* t*-test.

To gain further insights into the impact of elevated iron levels on bone microarchitecture in adult mice, we exposed 12-week-old C57BL/6J mice to the IRD for 6 or 12 weeks (Fig. 3A), aiming to comprehensively assess the effects of prolonged high iron levels on bone structure. Mice fed the IRD for either 6 or 12 weeks exhibited increased levels of hepatic iron, serum iron, unsaturated iron binding capacity, transferrin saturation, mean corpuscular volume, and mean corpuscular hemoglobin compared to the CD group (Suppl. Table 4). Interestingly, only the mice fed 6 weeks with an IRD showed increased values of red blood cells, hemoglobin, and hematocrit (Suppl. Table 4), indicating that the IRD successfully influenced iron metabolism and transport within the mice. Despite these observed changes in hematological and systemic iron parameters, the bone microarchitecture at the axial or appendicular skeleton (Fig. 3B–G, Table 1) and bone turnover markers in serum remained unaffected (Table 1). Collectively, these findings indicate that dietary iron overload in C57BL/6J mice does not affect bone health during skeletal development or homeostasis.

**Table 1:** Comparison of the bone microstructure of the femur and fourth lumbar vertebra (L4) of C57BL/6J adult male mice fed a control diet (CD) or an iron-rich diet (IRD) for 6 and 12 weeks.

	6 weeks of diet		12 weeks of diet	
	CD (n = 7)	IRD (n = 8)	CD (n = 7)	IRD (n = 8)
µCT parameter femur	Trabecular			
Tb. N (1/mm)	4.88 ± 0.13	4.96 ± 0.28	4.71 ± 0.44	4.67 ± 0.22
Tb. Th (µm)	0.050 ± 0.006	0.049 ± 0.005	0.049 ± 0.006	0.052 ± 0.003
Tb. Sp (mm)	0.199 ± 0.007	0.196 ± 0.013	0.207 ± 0.022	0.209 ± 0.011
BMD (g/cm^2^)	190.90 ± 34.04	196.77 ± 26.94	193.35 ± 40.58	211.08 ± 23.48
	Cortical			
Ct. Th (µm)	0.188 ± 0.013	0.186 ± 0.009	0.183 ± 0.011	0.190 ± 0.010
Ct. BMD (g/cm^2^)	993.56 ± 32.74	1004.90 ± 19.99	1032.27 ± 32.29	1028.01 ± 24.14
µCT parameter L4	Trabecular			
Tb. N (1/mm)	4.67 ± 0.39	4.57 ± 0.45	4.57 ± 0.35	4.56 ± 0.35
Tb. Th (µm)	0.053 ± 0.005	0.051 ± 0.004	0.051 ± 0.004	0.054 ± 0.002
Tb. Sp (mm)	0.212 ± 0.021	0.213 ± 0.025	0.215 ± 0.018	0.215 ± 0.015
BMD (g/cm^2^)	251.50 ± 19.27	238.65 ± 32.31	247.65 ± 29.83	262.20 ± 18.75
Serum marker				
PINP (ng/ml)	56.64 ± 9.36	78.15 ± 26.37	55.13 ± 18.95	51.03 ± 20.82
CTX-I (ng/ml)	16.56 ± 3.82	16.64 ± 3.67	16.22 ± 1.60	15.08 ± 2.67

*BV/TV* trabecular bone volume to total volume fraction, *Tb.N* trabecular number, *Tb.Th* trabecular thickness, *Tb.Sp* trabecular separation, *BMD* bone mineral density, *Ct* cortical, *PINP* procollagen type I N-terminal propeptide, *CTX-I* C-terminal telopeptide of type I collagen. Data is presented as mean ± SD. Statistics were calculated using Student’s *t*-test.

### Dietary iron overload caused bone loss in male 129/Sv mice exposed to excess iron in prenatal stage

**Figure 4:**
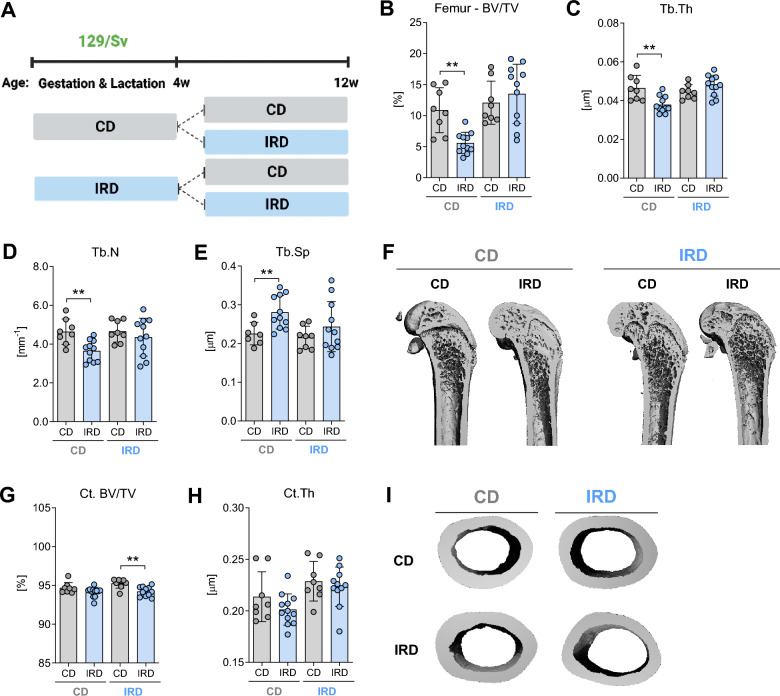
Trabecular and cortical bone morphology of the femora of 129/Sv mice fed a control diet or an iron-rich diet after prenatal exposure or not to excess iron. (**A**) Mice living in parental cages were fed one of two diets: a control diet (CD) or an iron-rich diet (IRD). This dietary regimen began three weeks before pregnancy and continued throughout gestation, with the offspring receiving the same diet as their mothers (either CD or IRD). At 4 weeks of age, mice from each dietary group were divided in one group receiving CD, while the other group receive the IRD until they reached 12 weeks of age. Trabecular bone parameters of (**B**) bone volume per total volume (BV/TV), (**C**) trabecular thickness (Tb. Th), (**D**) trabecular number (Tb. N), and (**E**) trabecular separation (Tb. Sp), were evaluated on the distal femur. (**F**) Representative 3D rendering of trabecular bone from the femora. Cortical bone parameters including (**G**) cortical bone volume (Ct. BV/TV) and (**H**) cortical thickness (Ct.Th), were evaluated at the femoral mid-shaft. (**I**) Representative 3D rendering of mid-diaphysis cortical bone from the femora. Data are presented as mean ± SD (n = 8–12). Each symbol represents an individual animal. Statistics were calculated using Student’s* t*-test. **P < 0.01.

To investigate the effects of excess iron exposure during pregnancy on bone characteristics in postnatal 129/Sv mice, we used a continuous iron exposure approach that started during gestation and continued throughout the lifespan. In this model, parental mice were assigned to one of two diets: CD or an IRD. This dietary regimen began three weeks before pregnancy and continued throughout gestation, with the offspring receiving the same diet as their mothers. At 4 weeks of age, mice from each dietary group were randomly divided into two subgroups: one group receiving CD, while the other group received the IRD until they reached 12 weeks of age (see Fig. 4A).

Mice exposed to a CD diet during both gestation and lactation, followed by a switch to an IRD, showed hepatic iron overload and increased *Hamp* mRNA expression (Suppl. Table 5). Conversely, mice exposed to an IRD diet during gestation and lactation, whether they maintained that diet or switched to a control diet later, did not display further elevations in hepatic iron content (Suppl. Table 5). Notably, no changes in iron accumulation were observed in bone tissue from the mice in any of the dietary groups (Suppl. Table 5). Additionally, both groups of mice fed an IRD experienced a significant decrease in red blood cell counts and a significant increase in mean corpuscular volume. However, reduced hemoglobin and hematocrit levels were observed only in mice from the CD group when they were fed an IRD (Suppl. Table S6). Concerning the influence of iron on the mRNA expression of iron related genes, no changes were observed in mice exposed to a CD and switched to an IRD. In contrast, mice exposed to an IRD during gestation and maintained in the same diet exhibited a decrease in the gene expression of *Tfr1* and *Slc40a1* (Suppl. Fig. 1B,C). The mRNA expression *Hfe* and *Hamp*, however, remained unaffected (Suppl. Fig. 1A,D).

**Figure 5:**
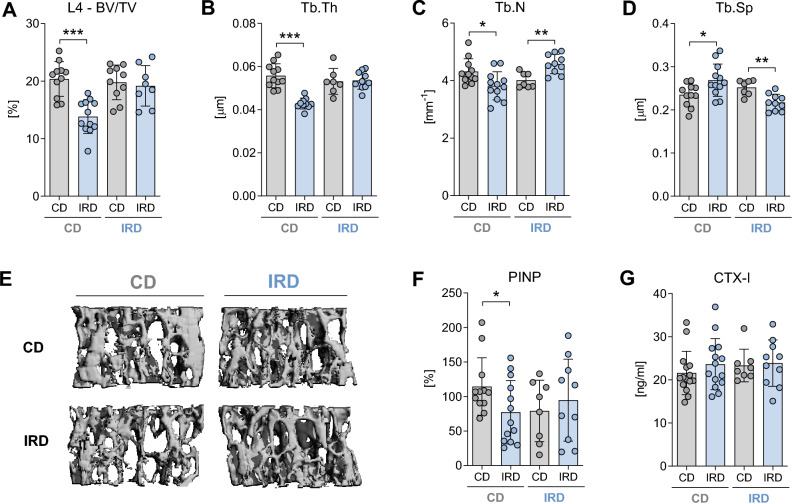
Trabecular bone phenotype of the L4 vertebra of 129/Sv mice fed a control diet or an iron-rich diet after prenatal exposure or not to excess iron. (**A**) Mice living in parental cages were fed one of two diets: a control diet (CD) or an iron-rich diet (IRD). This dietary regimen began three weeks before pregnancy and continued throughout gestation, with the offspring receiving the same diet as their mothers (either CD or IRD). At 6 weeks of age, mice from each dietary group were divided in one group receiving CD, while the other group receive the IRD until they reached 12 weeks of age. Trabecular bone parameters including (**A**) bone volume per total volume (BV/TV), (**B**) trabecular thickness (Tb. Th), (**C**) trabecular number (Tb. N), and (**D**) trabecular separation (Tb. Sp) were evaluated at L4 vertebral body. (**E**) Representative 3D rendering of trabecular bone from the L4 vertebra. Serum levels of (**F**) pro-collagen type 1 N-terminal peptide (PINP) and (**G**) C-terminal telopeptide of type I collagen (CTX-I) were also measured. Data are presented as mean ± SD (n = 10–14 per group). Each symbol represents an individual animal. *P < 0.05, **P < 0.01, ***P < 0.001.

Furthermore, assessment of the impact of excess iron on bone microarchitecture through µCT scans of the distal femur demonstrate that only mice from the CD group, who were fed an IRD after weaning, exhibited a reduction in trabecular bone volume (Fig. 4B). Additionally, trabecular thickness and numbers were significantly reduced (Fig. 4C,D), leading to increased trabecular separation (Fig. 4E,F). Moreover, mice from the CD group fed an IRD did not display significant reductions in cortical bone volume, although cortical thickness tended to be reduced (Fig. 4G,H). In contrast, cortical bone volume in mice from the IRD fed an IRD was significantly reduced when compared to mice on a CD. Representative images are depicted in Fig. 4I. At the fourth vertebral body, a noticeable decline in trabecular bone volume was observed in mice from the CD group that were fed an IRD diet when compared to those on a CD diet (Fig. 5A,E). In parallel, there were consistent reductions in trabecular thickness and the number of trabeculae (Fig. 5B,C), resulting in a significant increase in trabecular separation (Fig. 5D). Conversely, mice from the IRD group, who continued to consume an IRD after weaning, exhibited opposing trends in trabecular parameters. Both trabecular number and trabecular separation parameters showed significant changes in the opposite direction (Fig. 5C,D), although these alterations did not ultimately affect bone volume or trabecular thickness (Fig. 5A,E). Representative images are presented in Fig. 5E.

To evaluate the potential impact of an imbalance between bone resorption and bone formation processes on bone mass, we examined plasma levels of bone remodeling markers. Notably, the levels of the bone formation marker PINP were significantly lower in the serum of mice from the CD group that were fed an IRD diet after weaning, in comparison to mice maintained on a CD diet (Fig. 5F). No significant changes were observed in the circulating levels of CTX-I in any of the dietary groups analyzed (Fig. 5G). In addition, dynamic histomorphometry assessment at the L4-vertebra from mice of the CD group that were transitioned to an IRD diet showed no alterations in the bone remodeling parameters or in the mRNA expression in bone tissue of osteoblast and osteoclast genes in comparison to those maintained on a CD (Suppl. Fig. 6).

Taken together, these results suggest that (acute) dietary iron overload in 129/Sv mice leads to reduced bone density, while on the other hand, excessive iron exposure from the fetal stage to adulthood in mice protects mice from iron overload-induced trabecular bone loss, while cortical bone is negatively affected.

## Discussion

Osteoporosis and an increased incidence of fractures commonly occur in disorders associated with iron accumulation, such as hereditary hemochromatosis as well as in thalassemia, sickle cell disease, and myelodysplastic syndromes or even post-menopause^6,7,17,26^. However, it remains uncertain whether these manifestations are primarily due to the direct impact of iron on bone or if they result from concurrent underlying comorbidities that could potentially influence bone metabolism.

Many studies have explored the relationship between iron overload and changes in bone health. These investigations have been conducted both, in vitro using primary cultures and bone cell lines exposed to different sources of iron, and in vivo, using rodent animal models (revised in^4^). Iron supplementation has also been employed through various forms, such as iron dextran, ferric ammonium citrate, ferric nitriloacetate, or a diet enriched with 1–2.5% carbonyl iron. However, it is important to note that not all studies have consistently demonstrated effects on bone metabolism and that in particular the induction of in vivo iron loading appears to be influenced by factors such as duration of treatment, application form, sex, age, the use of littermates and the mouse strain investigated^3,16,18,19,27^.

In this study, we sought to analyze the effects of dietary iron overload on bone microarchitecture in two commonly used inbred strains, 129/Sv and C57BL/6J, to gain insights into the susceptibility of these strains to iron-induced bone alterations. We employed a comprehensive approach, examining the impact of iron overload at different stages of development, from fetal age to adult mice, for different durations, and assessed various bone parameters at both trabecular and cortical sites. Our model of dietary iron overload recapitulated essential features of clinical iron overload^19,28,29^. Initially, we focused on 129/Sv mice, which are frequently utilized in studies related to iron metabolism. Additionally, we conducted an examination of C57BL/6J mice, a strain widely preferred as a background strain for genetically modified mice^30^. While 129/Sv mice exposed to an IRD presented trabecular and cortical bone loss, we did not observe any significant bone alterations in C57BL/6J mice at any stage analyzed, despite the presence of elevated hepatic iron parameters. The observations in C57BL/6J are in contrast with previous reports using parenteral iron-dextran as a model of iron supplementation, which demonstrated bone loss in young mice (4 weeks old)^27^ and growing mice (8 weeks old) when higher doses of parenteral iron-dextran were administered (0.5 or 1.0 g/kg)^15,17^. Interestingly, when lower doses of iron dextran (0.1 g/kg)^17^ or ferric ammonium citrate (0.04 g/kg)^18^ or dietary iron (1% carbonyl iron)^19^ were used in ICR outbred or C57BL/6 mice, all of which caused systemic iron overload, the effect on bone was minimal or even resulted in bone gain. In line with this, other studies assessing the effects of iron in postmenopausal osteoporosis using ovariectomized ICR mice have shown a significant additional decrease in bone formation and resorption activities only when administering iron-dextran (0.04 g/kg) or ferric ammonium citrate (0.1 g/kg)^3,18^, indicating that iron affected bone mass on the top of estrogen-mediated bone loss. Taken together, our findings suggest that 129/Sv mice are susceptible to iron-induced bone loss and are thus more suitable for investigating the interaction between iron and bone compared to C57BL/6J mice.

Our study shows that dietary iron supplementation in both strains led to hepatic iron overload and increased *Hamp* mRNA expression, at levels seen in other experimental settings^31,32^. However, this alone was insufficient to induce bone loss under normal conditions in C57BL/6J, implying that the bone could adapt to prevent cell damage caused by iron overload, as the iron content in bone/bone marrow tissues remains unchanged. Moreover, previous reports have indicated that C57BL/6J mice exhibit unique iron characteristics primarily associated with differences in iron absorption capacity on duodenal epithelial cells and iron regulation at the tissue and cellular levels through the expression of hepatic transferrin receptors and ferritin, suggesting that this strain may have a lower capacity for iron uptake and storage in the liver compared to other inbred strains like Balb/c mice^33^. Furthermore, previous reports have indicated that iron accumulation and its effect can vary among different mouse strains, showing that strain differences strongly influence hepatic iron accumulation resulting from an iron-supplemented diet^34^. As an illustration, 129/Sv mice that exhibit higher basal iron levels seem to accumulate more iron than other strains. This implies that additional genes within this strain might be associated with iron metabolism and could play a role in the observed iron overload when given an IRD^34–36^. Notably, a study involving a large sample of mono- and dizygotic twins, illustrated that genetic factors also influence iron metabolism in humans^37^.

In addition, the fast rate of iron uptake through parenteral administration in contrast to the gradual absorption associated with dietary intake may play a significant role. Potentially, the slower uptake of iron via the diet allows the bone to adapt and cope with the increased amounts of iron. Alongside this, there are varying patterns of iron distribution^25^. While heavy iron deposits are observable in the bone marrow and trabeculae of mice receiving injections^17^, mice fed an IRD exhibit reduced to no apparent iron deposition in bone^19^. Hence, it is plausible that C57BL/6J mice are relative resistant to iron loading and exhibit decreased or unchanged levels of iron accumulation in their bones, potentially mitigating the adverse effects linked to excessive iron presence^38,39^.

Because nutrients are important for proper (skeletal) development, we also examined the effects of continuous exposure to elevated iron levels from the fetal stage to adulthood on bone homeostasis in 129/Sv mice. These mice exhibited significantly elevated hepatic iron content, accompanied by changes in blood parameters. Importantly, mice fed a CD during fetal state until weaning and then switched to an IRD for 6 weeks demonstrated that iron overload resulted in reduced trabecular bone density with modest effects on cortical bone structure. Interestingly, no bone loss occurred when mice received an IRD throughout their entire lifespan (starting in utero). While the reasons behind these differing responses to the same cumulative iron dosage require further elucidation, one hypothesis is that bone tissue might use adaptation mechanisms to counter the detrimental effects of excessive iron e.g. by adjusting the IRE/IRP system as reported in skeletal muscle^40^, triggering protective antioxidant responses^41^, or employing iron excretion via the feces as observed to confer protection against iron-triggered cardiac issues^42,43^. Of note, considering that many women take iron supplements during pregnancy, our data may indicate that this may not be harmful to the bone health of the offspring.

In conclusion, our study contributes to the understanding of the impact of iron overload on bone microarchitecture in different mouse strains. The findings underscore the intricate relationship between hereditable factors, iron metabolism, and bone health. These insights have implications for further research on iron-induced bone pathology and emphasize the importance of considering the genetic background when studying the effects of iron overload on skeletal health.

### Supplementary Information

Supplementary Figures.

Supplementary Tables.

## Data Availability

All data generated or analyzed during this study are included in this published article and its supplementary information files. This work did not generate new datasets.
