# Supplementary material for: Comparison of the effects of high dietary iron levels on bone microarchitecture responses in the mouse strains 129/Sv and C57BL/6J
Source: Sci Rep. 2024 Feb 28;14:4887. doi: 10.1038/s41598-024-55303-2 (PMC10902348; doi:10.1038/s41598-024-55303-2)
Supplement: Supplementary file 1 — Supplementary Figures. [file 41598_2024_55303_MOESM1_ESM.pdf]

Comparison of the effects of high dietary iron levels on bone microarchitecture responses in the mouse strains 129/Sv and C57BL/6J

Maria G. Ledesma-Colunga[1], Vanessa Passin[1], Maja Vujic Spasic[2],

Lorenz C. Hofbauer[1], Ulrike Baschant[1], Martina Rauner[1]*

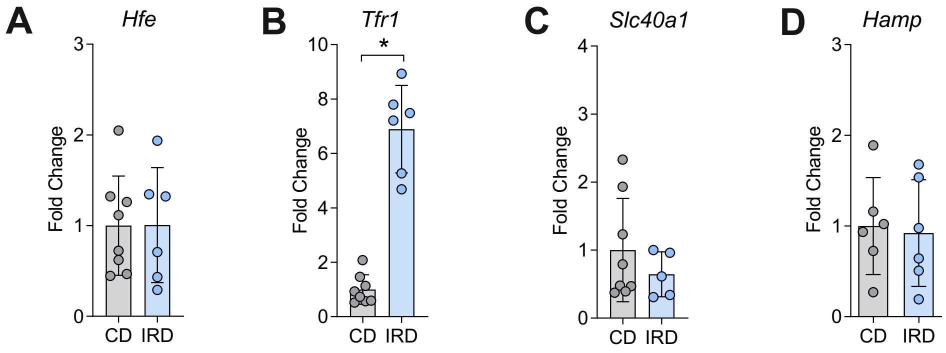

Supplementary Figure 1.

Supplementary Figure 1. Analysis of gene expression related to iron metabolism. mRNA levels were evaluated in the femoral shaft/bone marrow of 129/Sv mice fed a control diet or an iron-rich diet for 6 weeks. The examined genes include (**A)** *Hfe,* **(B)** *Tfr1*, **(C)** *Slc40a1*, and **(D)** *Hamp*. Data are presented as mean ± SD (n= 7-8 per group). Each symbol represents an individual animal. Statistics were calculated using Student´s *t*-test. *P < 0.05, **P < 0.01, ***P < 0.001.

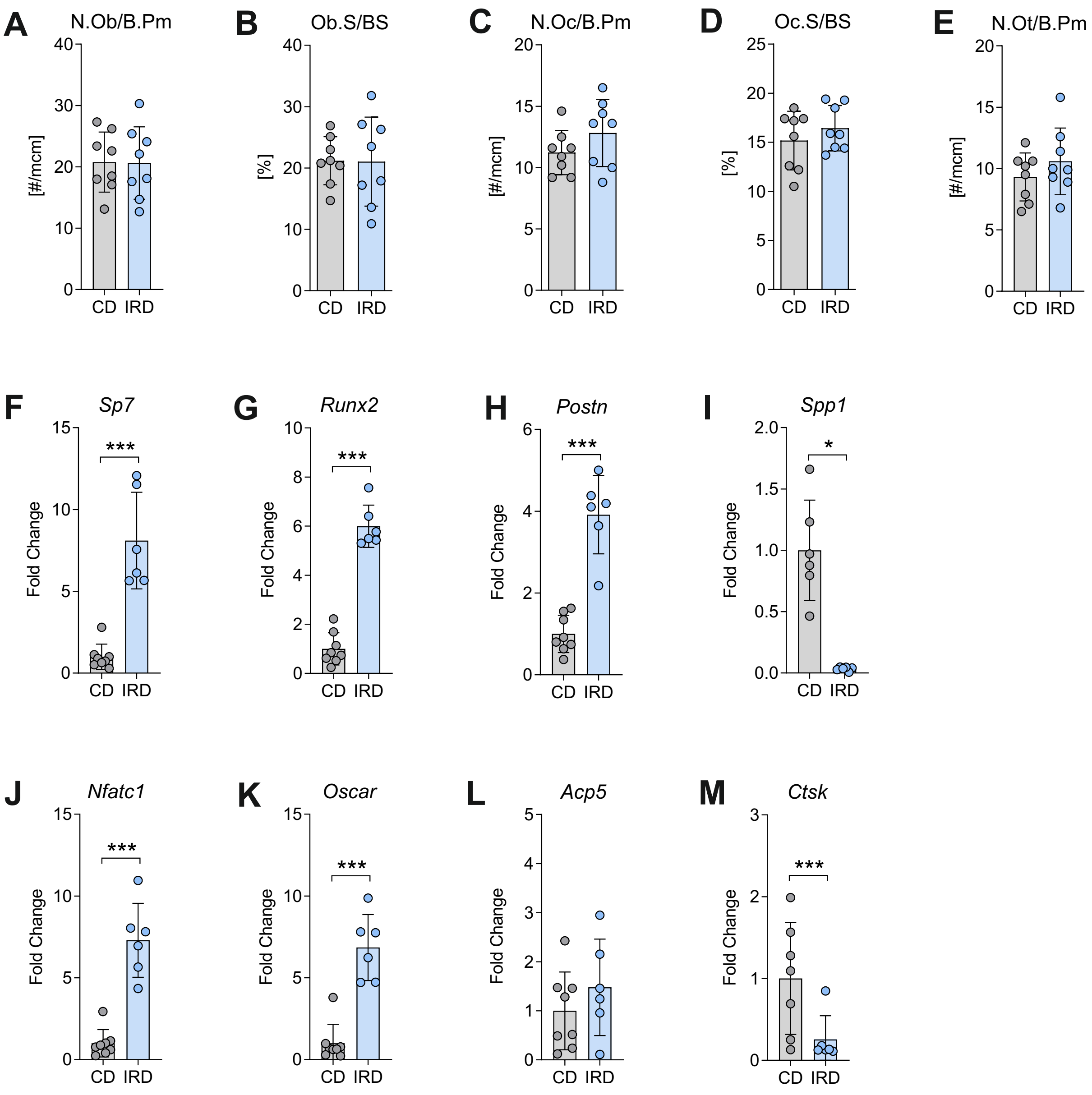

Supplementary Figure 2.

Supplementary Figure 2. Histomorphometric assessment and gene expression analysis of osteoblasts and osteoclasts. Static indices for osteoblast and osteoclast activity were determined in TRAP-stained sections of the L4 vertebra from 129/Sv mice subjected to either a control or iron-rich diet for a 6-week period. The parameters evaluated include **(A)** the number of osteoblasts per bone perimeter (N.Ob/B.Pm), **(B)** osteoblast surface per bone surface (Ob.S/BS), **(C)** number of osteoclasts per bone perimeter (N.Oc/B.Pm), **(D)** osteoclast surface per bone surface (Oc.S/BS), and **(E)** number of osteocytes per bone perimeter (N.Ot/B.Pm). Additionally, gene expression analysis of osteoblast and osteoclast markers in the femoral shaft/bone marrow is presented, including **(F)** *Sp7*, **(G)** *Runx2*, **(H)** *Postn*, **(I)** *Spp1*, **(J)** *Nfatc1*, **(K)** *Osca*r, **(L)** *Acp5*, and **(M)** *Ctsk*. Data are presented as mean ± SD (n= 7-8 per group). Each symbol represents an individual animal. Statistics were calculated using Student´s *t*-test. *P < 0.05, **P < 0.01, ***P < 0.001.

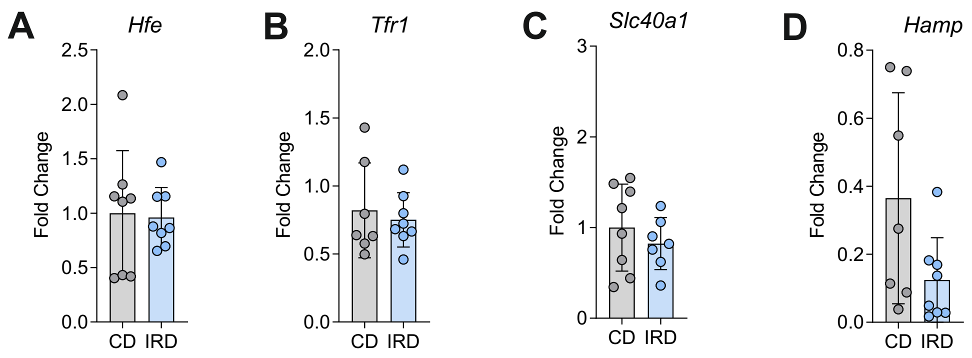

Supplementary Figure 3.

Supplementary Figure 3. Analysis of gene expression related to iron metabolism. mRNA levels were evaluated in the femoral shaft/bone marrow of young C57BL/6J mice fed a control diet or an iron-rich diet for 6 weeks. The examined genes include (**A)** *Hfe,* **(B)** *Tfr1*, **(C)** *Slc40a1*, and **(D)** *Hamp*. Data are presented as mean ± SD (n= 7-8 per group). Each symbol represents an individual animal. Statistics were calculated using Student´s *t*-test. *P < 0.05, **P < 0.01, ***P < 0.001.

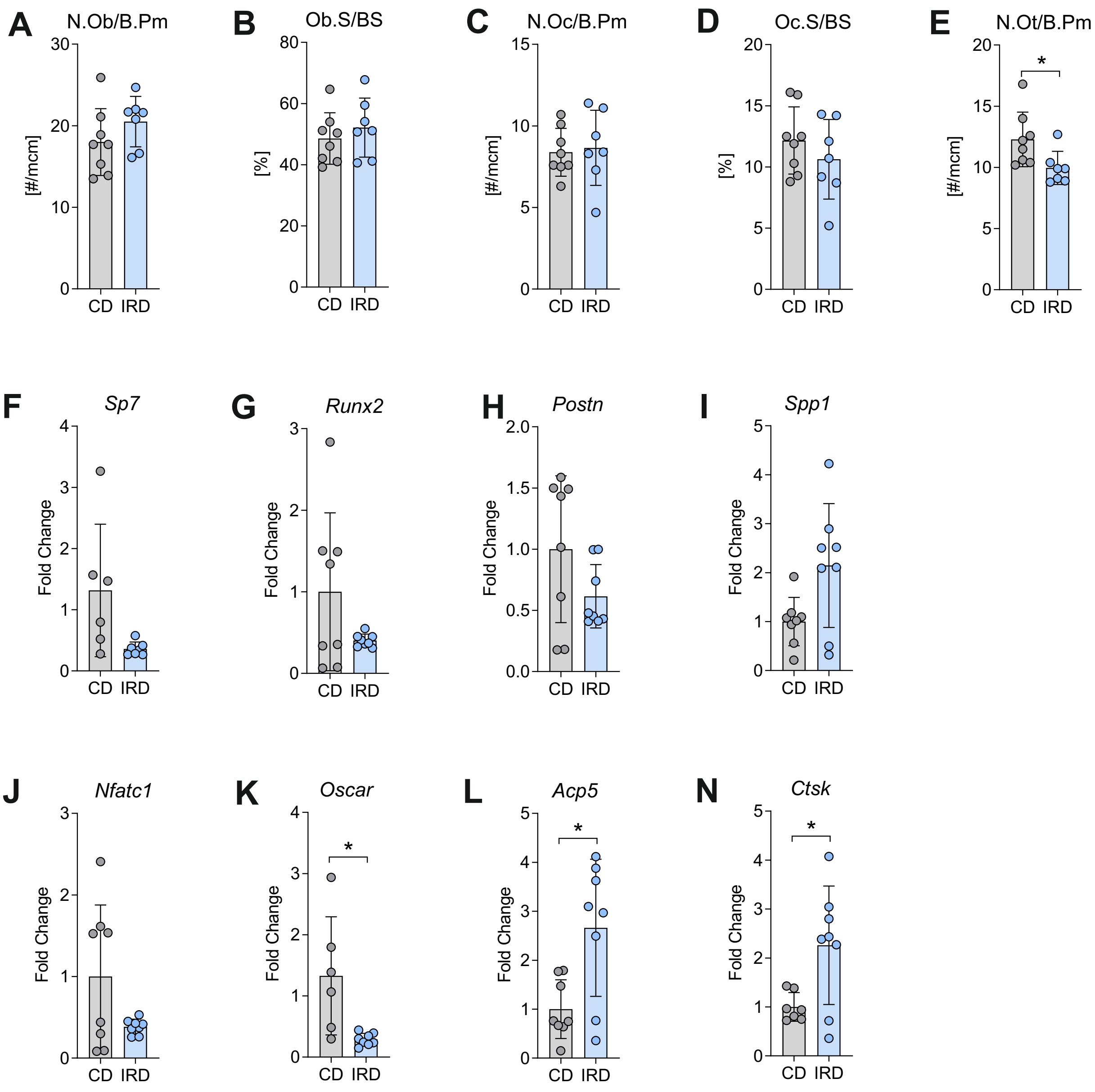

Supplementary Figure 4.

Supplementary Figure 4. Histomorphometric assessment and gene expression analysis of osteoblasts and osteoclasts. Static indices for osteoblast and osteoclast activity were determined in TRAP-stained sections of the L4 vertebra from young C57BL/6J mice subjected to either a control or iron-rich diet for a 6-week period. The parameters evaluated include **(A)** the number of osteoblasts per bone perimeter (N.Ob/B.Pm), **(B)** osteoblast surface per bone surface (Ob.S/BS), **(C)** number of osteoclasts per bone perimeter (N.Oc/B.Pm), **(D)** osteoclast surface per bone surface (Oc.S/BS), and **(E)** number of osteocytes per bone perimeter (N.Ot/B.Pm). Additionally, gene expression analysis of osteoblast and osteoclast markers in the femoral shaft/bone marrow is presented, including **(F)** *Sp7*, **(G)** *Runx2*, **(H)** *Postn*, **(I)** *Spp1*, **(J)** *Nfatc1*, **(K)** *Osca*r, **(L)** *Acp5*, and **(M)** *Ctsk*. Data are presented as mean ± SD (n= 7-8 per group). Each symbol represents an individual animal. Statistics were calculated using Student´s *t*-test. *P < 0.05, **P < 0.01, ***P < 0.001.

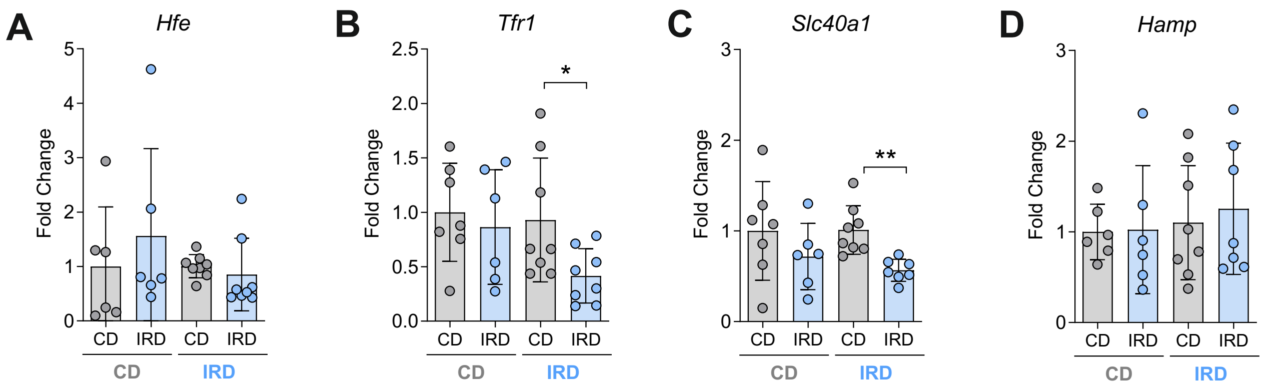

Supplementary Figure 5.

Supplementary Figure 5. Analysis of gene expression related to iron metabolism. Mice living in parental cages were fed one of two diets: a control diet (CD) or an iron-rich diet (IRD). This dietary regimen began three weeks before pregnancy and continued throughout gestation, with the offspring receiving the same diet as their mothers (either CD or IRD). At 4 weeks of age, mice from each dietary group were further divided, with one subgroup continuing on the CD, while the other transitioned to the IRD until reaching 12 weeks of age. mRNA levels were assessed in the femoral shaft/bone marrow of these mice, focusing on the genes (**A)** *Hfe,* **(B)** *Tfr1*, **(C)** *Slc40a1*, and **(D)** *Hamp*. Data are presented as mean ± SD (n= 7-8 per group). Each symbol represents an individual animal. Statistics were calculated using Student´s *t*-test. *P < 0.05, **P < 0.01, ***P < 0.001.

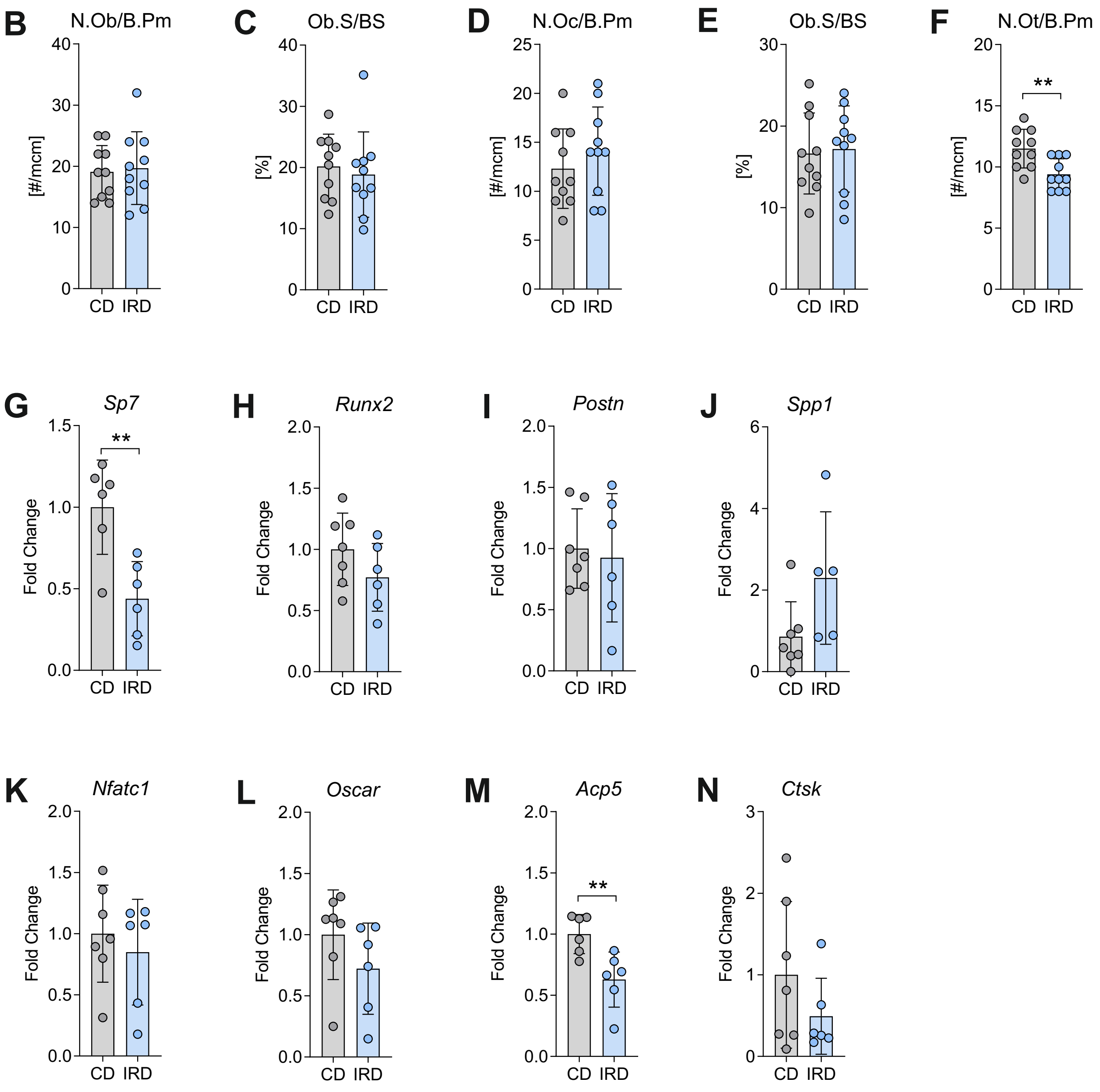

Supplementary Figure 6.

Supplementary Figure 6. Histomorphometric assessment and gene expression analysis of osteoblasts and osteoclasts. Static indices for osteoblast and osteoclast activity were determined in TRAP-stained sections of the L4 vertebra from mice fed a control diet (CD) throughout gestation. At 4 weeks of age, the offspring from this group were subdivided, with one subgroup continuing on the CD, while the other transitioned to the iron-rich diet (IRD) until reaching 12 weeks of age. The parameters evaluated include **(A)** the number of osteoblasts per bone perimeter (N.Ob/B.Pm), **(B)** osteoblast surface per bone surface (Ob.S/BS), **(C)** number of osteoclasts per bone perimeter (N.Oc/B.Pm), **(D)** osteoclast surface per bone surface (Oc.S/BS), and **(E)** number of osteocytes per bone perimeter (N.Ot/B.Pm). Additionally, gene expression analysis of osteoblast and osteoclast markers in the femoral shaft/bone marrow is presented, including **(F)** *Sp7*, **(G)** *Runx2*, **(H)** *Postn*, **(I)** *Spp1*, **(J)** *Nfatc1*, **(K)** *Osca*r, **(L)** *Acp5*, and **(M)** *Ctsk*. Data are presented as mean ± SD (n= 7-8 per group). Each symbol represents an individual animal. Statistics were calculated using Student´s *t*-test. *P < 0.05, **P < 0.01, ***P < 0.001.
