# Supplementary material for: Comparison of the effects of high dietary iron levels on bone microarchitecture responses in the mouse strains 129/Sv and C57BL/6J
Source: Sci Rep. 2024 Feb 28;14:4887. doi: 10.1038/s41598-024-55303-2 (PMC10902348; doi:10.1038/s41598-024-55303-2)
Supplement: Supplementary file 2 — Supplementary Tables. [file 41598_2024_55303_MOESM2_ESM.docx]

**Comparison of the effects of high dietary iron levels on the bone microarchitecture responses in two inbred mouse strains: 129/Sv and C57BL/6J**

Maria G. Ledesma-Colunga^1^, Vanessa Passin^1^, Maja Vujic Spasic^2^,

Lorenz C. Hofbauer^1^, Ulrike Baschant^1^, Martina Rauner^1^*

**Table S1.** Oligonucleotide sequences used for quantitative real-time PCR.

| ***Gene*** | ***Sequence (5´-3´)*** | |
| --- | --- | --- |
| *Actb* | s: ATCTGGCACCACACCTTCT | as: GGGGTGTTGAAGGTCTC AAA |
| *Hfe* | s: CACCGTCTGTGCCATCTTCTT | as: ACATAGCCACCCATGGTTCCT |
| *Hamp* | s: GCACCACCTATCTCCATCAAC | as: AGAAGATGCAGATGGGGAAG |
| *Fpn* | s: TGTCAGCCTGCTGTTTGCAGGA | as: TCTTGCAGCAACTGTGTCACCG |
| *Tfr1* | s: TAAATTCCCCGTTGTTGAGG | as: ATGACTGAGATGGCGGAAAC |
| *Sp7* | s: CTTCCCAATCCTATTTGCCGTTT | as: CGGCCAGGTTACTAACACCAATCT |
| *Runx2* | s: AAATGCCTCCGCTGTTATGA | as: GCTCCGGCCCACAAATCT |
| *Postn* | s: CCACTCAGCACTACTCCGATG | as: TCTCCCAAGCCTCGTTACTC |
| *Spp1* | s: TGAAAGTGACTGATTCTGGCA | as: GGACGATTGGAGTGAAAGTGT |
| *Nfatc1* | s: GTTCCTTCAGCCAATCATCC | as: GGAGGTGATCTCGATTCTCG |
| *Oscar* | s: TGGCGGTTTGCACTCTTCA | as: GATCCGTTACCAGCAGTTCCAGA |
| *Acp5* | s: ACTTGCGACCATTGTTAGCC | as: AGAGGGATCCATGAAGTTGC |
| *Ctsk* | s: AGTGGTTCAGAAGATGACGGGAC | as: TCTTCAGAGTCAATGCCTCCGTTC |

**Table S2.** Hematological and systemic iron parameters in 129/Sv male mice fed a control diet (CD) or an iron-rich diet (IRD) for 6 weeks.

| **129/Sv** | ***6 weeks of diet*** | |
| --- | --- | --- |
| ***Parameter*** | **CD (n=8)** | **IRD (n=8)** |
| **RBC (10^9/L)** | 10.49 ± 0.71 | 10.18 ± 0.43 |
| **Hb (mmol/L)** | 10.09 ± 0.62 | 9.79 ± 0.33 |
| **Hct (L/L)** | 0.55 ± 0.05 | 0.51 ± 0.02 |
| **MCV (fL)** | 52.93 ± 3.56 | 50.25 ± 1.38 |
| **MCH (fmol)** | 0.97 ± 0.02 | 0.96 ± 0.02 |
| **MCHC (mmol/L)** | 18.26 ± 1.29 | 19.15 ± 0.26 |
| **Serum Fe (μg/dL)** | 255.96 ± 41.03 | 277.17 ± 34.08 |
| **UIBC (µg/dL)** | 190.34 ± 64.12 | 165.32 ± 18.85 |
| **TfSat (%)** | 58.21 ± 7.44 | 62.53 ± 3.69 |
| **Liver Fe (µg/g)** | 203.51 ± 27.77 | 1189.33 ± 170.48*** |
| **Bone/Bone Marrow Fe (µg/g)** | 42.62 ± 13.78 | 49.22 ± 10.60 |
| **Liver *Hamp* mRNA** | 1.00 ± 0.68 | 7.30 ± 1.65*** |

RBC: red blood cells; Hb: hemoglobin; Hct: hematocrit; MCV: mean corpuscular volume; MCH: mean corpuscular hemoglobin; MCHC: mean corpuscular hemoglobin concentration; Serum Fe: serum iron; UIBC: unsaturated iron binding capacity; TfSat: transferrin saturation; Liver Fe, Bone Fe: non-heme iron content in dried tissue. Data is presented as mean ± SD. Statistics were calculated using Student´s *t*-test. ***P<0.001.

**Table S3.** Hematological and systemic iron parameters in young C57BL/6J male mice fed a control diet (CD) or an iron-rich diet (IRD) for 6 weeks.

| **C57BL/6J** | ***6 weeks of diet*** | | |
| --- | --- | --- | --- |
| ***Parameter*** | **CD (n=8-16)** | **IRD (n=8-16)** |  |
| **RBC (10^9/L)** | 10.29 ± 0.45 | 9.44 ± 0.46 | |
| **Hb (mmol/L)** | 9.51 ± 0.45 | 9.08 ± 0.47 | |
| **Hct (L/L)** | 0.50 ± 0.02 | 0.49 ± 0.03 | |
| **MCV (fL)** | 48.56 ± 1.06 | 51.60 ± 0.47 | |
| **MCH (fmol)** | 0.92 ± 0.01 | 0.96 ± 0.02 | |
| **MCHC (mmol/L)** | 19.03 ± 0.62 | 18.63 ± 0.23 | |
| **Serum Fe (μg/dL)** | 250.14 ± 70.53 | 292.03 ± 159.43 | |
| **UIBC (µg/dL)** | 144.86 ± 39.72 | 70.40 ± 40.79** | |
| **TfSat (%)** | 66.38 ± 13.21 | 89.10 ± 7.754 | |
| **Liver Fe (µg/g)** | 153.08 ± 59.76 | 1102.06 ± 405.32*** | |
| **Bone/Bone Marrow Fe (µg/g)** | 65.34 ± 27.57 | 90.60 ± 39.23 | |
| **Liver *Hamp* mRNA** | 1.00 ± 0.41 | 4.98 ± 1.58*** | |

RBC: red blood cells; Hb: hemoglobin; Hct: hematocrit; MCV: mean corpuscular volume; MCH: mean corpuscular hemoglobin; MCHC: mean corpuscular hemoglobin concentration; Serum Fe: serum iron; UIBC: unsaturated iron binding capacity; TfSat: transferrin saturation; Liver Fe, Bone Fe: non-heme iron content in dried tissue. Data is presented as mean ± SD. Statistics were calculated using Student´s *t*-test. **P<0.01, ***P<0.001.

**Table S4.** Hematological and systemic iron parameters in adult C57BL/6J male mice fed a control diet (CD) or an iron-rich diet (IRD) for 6 and 12 weeks.

| **C57BL/6J** | ***6 weeks of diet*** | | ***12 weeks of diet*** | |
| --- | --- | --- | --- | --- |
| ***Parameter*** | **CD (n= 7)** | **IRD (n= 8)** | **CD (n= 7)** | **IRD (n= 8)** |
| **RBC (10^12^/L** | 9.42 ± 0.25 | 9.90 ± 0.43* | 10.00 ± 0.31 | 9.61 ± 0.98 |
| **Hb (mmol/L)** | 8.83 ± 0.31 | 9.81 ± 0.38** | 9.30 ± 0.23 | 9.68 ± 0.90 |
| **Hct (L/L)** | 0.47 ± 0.01 | 0.52 ± 0.02** | 0.49 ± 0.01 | 0.52 ± 0.05 |
| **MCV (fL)** | 49.41 ± 0.56 | 52.50 ± 0.35** | 49.56 ± 0.51 | 53.98 ± 1.02** |
| **MCH (fmol)** | 0.94 ± 0.03 | 0.99 ± 0.01** | 0.93 ± 0.01 | 1.01 ± 0.02** |
| **MCHC (fmol/L)** | 18.97 ± 0.36 | 18.89 ± 0.20 | 18.80 ± 0.23 | 18.68 ± 0.30 |
| **Serum Fe (μg/dL)** | 101.30 ± 15.10 | 217.79 ± 32.31** | 121.04 ± 17.65 | 177.72 ± 10.84** |
| **UIBC (µg/dL)** | 233.89 ± 40.04 | 85.00 ± 17.95** | 230.48 ± 31.81 | 64.87 ± 23.84** |
| **TfSat (%)** | 30.63 ± 5.32 | 72.11 ± 4.57** | 34.62 ± 5.20 | 73.92 ± 8.63** |
| **Liver Fe (µg/g)** | 247.85 ± 42.76 | 1011.48 ± 0.392*** | 204.39 ± 40.25 | 1369.62 ± 399.29*** |

RBC: red blood cells; Hb: hemoglobin; Hct: hematocrit; MCV: mean corpuscular volume; MCH: mean corpuscular hemoglobin; MCHC: mean corpuscular hemoglobin concentration; Serum Fe: serum iron; UIBC: unsaturated iron binding capacity; TfSat: transferrin saturation. Liver Fe, non-heme iron content in dried tissue. Data is presented as mean ± SD. Statistics were calculated using Student´s *t*-test. *P<0.05, **P<0.01, ***P<0.001.

**Table S5.** Hematological and systemic iron parameters in 129/Sv male mice fed a control diet (CD) or and iron-rich diet (IRD) during gestation and lactation, followed by either a CD or IRD for 6 weeks.

|  | ***CD during gestation and lactation*** | | ***IRD during gestation and lactation*** | |
| --- | --- | --- | --- | --- |
| **129/Sv** | ***6 weeks of diet*** | | ***6 weeks of diet*** | |
| ***Parameter*** | **CD (n=8-14)** | **IRD (n=8-14)** | **CD (n=8-11)** | **IRD (n=8-11)** |
| **RBC (10^9/L)** | 11.62 ± 2.45 | 9.73 ± 0.62* | 10.24 ± 1.01 | 8.85 ± 1.27* |
| **Hb (mmol/L)** | 11.34 ± 2.30 | 9.66 ± 0.50* | 9.49 ± 0.97 | 8.76 ± 1.20 |
| **Hct (L/L)** | 0.60 ± 0.12 | 0.51 ± 0.03* | 0.51 ± 0.06 | 0.47 ± 0.07 |
| **MCV (fL)** | 51.18 ± 1.15 | 52.26 ± 1.16* | 49.89 ± 1.30 | 52.71 ± 1.72** |
| **MCH (fmol)** | 0.98 ± 0.04 | 1.00 ± 0.03 | 0.93 ± 0.02 | 1.00 ± 0.03** |
| **MCHC (mmol/L)** | 19.06 ± 0.33 | 19.03 ± 0.46 | 18.59 ± 0.34 | 18.83 ± 0.36 |
| **Serum Fe (μg/dL)** | 236.62 ± 50.85 | 259.79 ± 44.11 | 198.73 ± 75.56 | 204.05 ± 47.07 |
| **UIBC (µg/dL)** | 379.11 ± 101.81 | 448.63 ± 134.99 | 525.83 ± 54.75 | 474.94 ± 108.45 |
| **TfSat (%)** | 39.08 ± 9.06 | 40.98 ± 13.64 | 30.58 ± 6.59 | 34.01 ± 7.66 |
| **Liver Fe (µg/g)** | 202.3 ± 91.49 | 780.67 ± 201.94*** | 192.12 ± 21.13 | 1154.31 ± 469.20*** |
| **Bone Fe (µg/g)** | 26.68 ± 11.88 | 31.00 ±28.21 | 20.10 ± 14.37 | 33.95 ± 12.85 |
| **Liver Hamp mRNA** | 1.00 ± 0.36 | 5.13 ± 1.47*** | 1.00 ± 0.73 | 6.19 ± 4.59** |

RBC: red blood cells; Hb: hemoglobin; Hct: hematocrit; MCV: mean corpuscular volume; MCH: mean corpuscular hemoglobin; MCHC: mean corpuscular hemoglobin concentration; Serum Fe: serum iron; UIBC: unsaturated iron binding capacity; TfSat: transferrin saturation; Liver Fe, Bone Fe: non-heme iron content in dried tissue. Data is presented as mean ± SD. Statistics were calculated using Student´s *t*-test. *****P<0.05, ******P<0.01, *******P<0.001.
